# The Public Health Crisis Conceptual Model: Historical Application to the World’s First Nuclear Bomb Test

**DOI:** 10.3390/socsci13040186

**Published:** 2024-03-25

**Authors:** Mary Pat Couig, Roberta Lavin, Heidi Honegger Rogers, Sara Bandish Nugent

**Affiliations:** College of Nursing, University of New Mexico, Albuquerque, NM 87131, USA

**Keywords:** public health crisis conceptual model, nuclear preparedness, radiation preparedness, nursing

## Abstract

**Background/purpose:**

The Public Health Crisis Conceptual Model was developed to identify and address healthcare and human services needs related to a disaster. The purpose of this study was to historically apply this model to the counties and populations most affected by the first nuclear test in 1945, with a focus on community and local priorities, and to further describe this model and validate its usefulness. If the model had been applied in 1945, what might have been different with respect to research, epidemiological studies, and reparations?

**Methods:**

A historical, descriptive case study approach was used, with a focus on community and local priorities.

**Results:**

While it was deemed necessary to maintain secrecy surrounding the Trinity test during wartime efforts, scientists and the military knew of the potential dangers of radioactive fallout. However, they neglected to inform exposed New Mexicans after the information about the nature of the bombings of Hiroshima and Nagasaki had been made public.

**Conclusions:**

Research and epidemiological studies could have been implemented years before they were. Resources were not and have not been distributed equitably to those exposed to fallout from the Trinity test site. Using the Public Health Crisis Conceptual Model will help ensure that community and local priorities are an integral component of future disaster-related research.

## 1. Introduction

**Figure 1.**
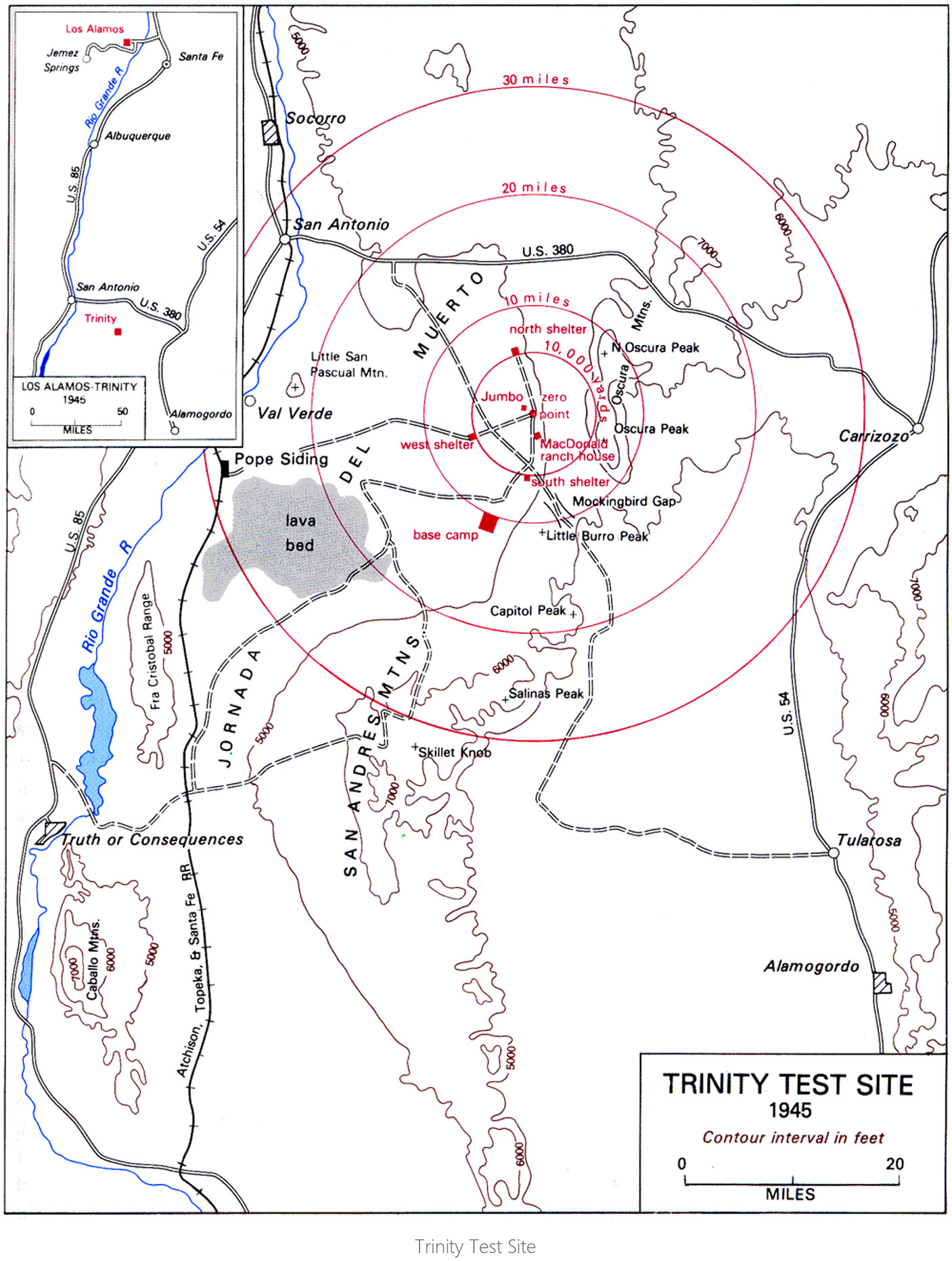
Trinity test site location and surrounding communities (Jones 1985, p. 656). Trinity Test Site. Reprinted from Vincent C. Jones, Manhattan: The Army and the Atomic Bomb

On 16 July 1945, at 05:29:45 Mountain Time, the first nuclear bomb was detonated at the Trinity test site, located in the Jornado del Muerto desert within the White Sands Proving Ground in New Mexico, United States of America (Figure 1). At the time of the blast, there were approximately 37,500 people living in the community and surrounding counties—Lincoln, Otero, Sierra, and Socorro (U.S. Census Bureau 1940). The military was concerned about the possibility of the bomb detonating, but with respect to planning for the communities most affected by the fallout, they undertook little planning and obtained no input from them due to the secretive nature of the test for military security reasons. Reports from observers as far as 200 miles away described explosions, bright lights, and falling ash that children thought was snow. In response to the explosion, the Alamogordo Air Base’s press release stated, “A remotely located ammunition magazine containing a considerable amount of high explosives and pyrotechnics exploded, but there was no loss of life or limb to anyone.” (US Air Force et al. n.d.) The world would not learn the extent of the “scientific and technical” advances in the development of nuclear energy for military use until after the bombs were dropped in Japan and the “Smyth” report on *Atomic Energy for Military Purposes* (Smyth 1945) was released in August 1945. It took another 40 years before data on the scope and impact of the Trinity fallout plume were published, and 60 years for the first report on the potential health impacts and routes of exposures to be analyzed and reported (Simon et al. 2021).

Of the nine countries with nuclear weapons (China, India, Israel, France, North Korea, Pakistan, Russia, United Kingdom, and United States, possessing an estimated total of 13,000 warheads) (Phelan 2022), two are at war with countries with no nuclear weapons while another has escalated its aggression in the Indo-Pacific Region (Lopez 2022). The United States’ National Security Strategy (The White House 2022) highlights the potential for nuclear events and the ongoing need for nuclear deterrence.

In addition to planning for the threat of a nuclear explosion, there is also a need to plan for potential threats from the 56 nuclear power plants in the United States, with a population of approximately 3 million people living within 10 miles of those plants (Federal Emergency Management Agency 2023). Globally, there are approximately 440 nuclear power reactors in 32 countries, and more than 50 countries use clean nuclear energy in over 220 research reactors; radioactive isotopes for health purposes are made by these reactors (World Nuclear Association 2023). Nuclear contamination from the air can spread a great distance from a detonation site, being dependent on wind direction and speed. Widespread land and water contamination can endanger water and food systems for extended periods of time (Simon et al. 2021) and can cause irreversible ecological damage, further impacting biodiversity (Mousseau 2021).

Nuclear explosions have multiple destructive pathways to cause damage in the short and long-term—blast, thermal and initial nuclear radiation, and fallout. In addition to the immediate damage at the epicenter of an explosion, electromagnetic and particulate radiation are released and the energy is transformed into “.…a fireball, blast/forces/waves, prompt radiation, light and heat (thermal energy) and delayed ionizing radiation.” (Radiation Emergency Medical Management 2023). Physiologic effects from these forces include the following: blast injuries to multiple systems; crush injuries; penetrating and blunt trauma; burn injuries to the eyes and skin; acute radiation syndrome and cancers from cellular changes. The review by Shigemura et al. (2021) on the psychological effects from the Fukushima disaster found outcomes of anxiety, non-specific distress, depression and post-traumatic stress disorder ranging from 8.3–65.1%. The number of people affected depends on the size and scope of the nuclear disaster or the detonation of the nuclear weapon and weather patterns and geography/location at the time of an event. In addition to direct exposure to nuclear explosions and immediate exposure to radioactive nuclear fallout, as well as destruction of land/buildings and infrastructure, nuclear disasters frequently result in long term contamination of water supplies, food, and soil for decades.

In New Mexico, the Department of Homeland Security and Emergency Management is responsible for preparing programs and plans to prevent, protect, mitigate, respond to and recover from a broad range of potential threats (New Mexico Department of Homeland Security and Emergency Management n.d.) including Hazardous Materials Emergency Preparedness (NM Department of Homeland Security and Emergency Management n.d.).

Disaster preparedness, response, and recovery have been previously studied using ecological models. These models examine how individuals and populations interact with multiple levels of their environment, and also depict the reciprocal influence between these levels (Glanz et al. 2015). One of the most well-known ecological theories was first developed by Bronfenbrenner (1979), which focused on human development.

Within the disaster context, a number of ecological models have been presented. The Eco-Social Trauma Intervention Model was developed to guide practice and service delivery, organize research, and inform public policy after a trauma has occurred (Gultekin et al. 2019). The Ecological Model of Disaster Management focuses on establishing systems and structures post-disaster (Beaton et al. 2008). Additionally, the Social Ecological Model of Disaster Resilience is framed from the perspective of the affected individual and/or individual community, rather than the disaster as a whole (Bell et al. 2021a, 2021b). The final framework, the Disaster Ecology Model (DEM), examines how the disaster type, magnitude, time dimension, and impact location affect communities and populations at different environmental contextual levels (Shultz et al. 2007). The DEM is the only ecological model that has been applied to a nuclear disaster. To accomplish this, Shultz et al. (2013) used the trauma signature analysis, which was based on the DEM, to identify evidence-based psychological risk factors caused by The Great East Japan Earthquake, which included an earthquake, tsunami, and nuclear accident.

Nuclear disasters are highly complex phenomena, and while each ecological model provides sufficient detail for certain aspects of disasters, none specifically focuses on the entire process from preparedness to recovery. Additionally, none of these models examines the physiological health outcomes of affected communities, which is imperative, since radiation exposure can have short and long term adverse health consequences.

**Figure 2.**
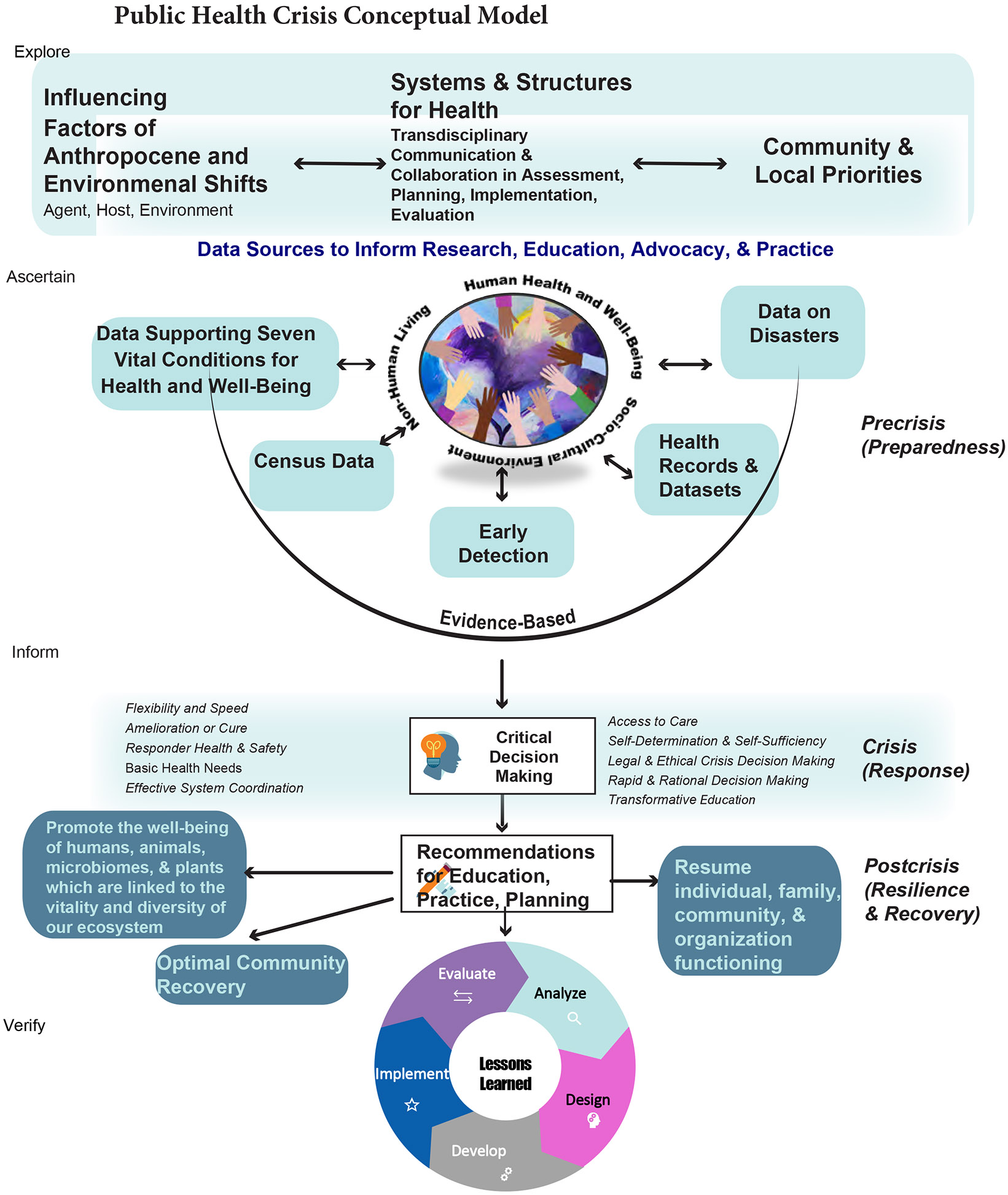
Public Health Crisis Conceptual Model (rev 2023).

The Public Health Crisis Conceptual Model (PHCCM) (Figure 2) was developed to address healthcare and human services needs related to a disaster (Lavin et al. 2023). Initially, it was designed as a human-centered analytic approach to assess the “as is” situation and develop a “to be” plan. This was achieved by adapting a crisis conceptual model and incorporating principles from social justice nursing and public health preparedness into a human-centered structured analytical approach (Lavin et al. 2019; Veenema et al. 2019). More recently, the Seven Vital Conditions of Health and Well-Being (Community Commons n.d.b) have been incorporated as part of the evidence base. The primary components include: (1) influencing factors, (2) systems and structures for health, (3) community and local priorities, (4) data sources, (5), critical decision-making, and (6) recommendations for education, practice, policy, and planning. We hypothesize that these components will improve disaster-related health outcomes in preparedness plans through advanced training, better access to healthcare, and improved disaster survivor support. Definitions for the model can be found in Table 1.

The PHCCM incorporates principles of the Planetary Health Education Framework (PHEF) (Faeron Guzman and Potter n.d.). Planetary health is inherently interdisciplinary, examining the ecological drivers of health, which include radioactive pollution. It also discusses mediating factors aimed at mitigating health harms, reducing exposures, enhancing screening, and promoting adaptation and resilience within the context of environmental damage/destruction. The PHEF provides a shared language and invites transdisciplinary partnerships across sectors to promote health equity and justice, reduce health harms, and mitigate the immediate causes of health hazards. Planetary health is a way of thinking about human health in the Anthropocene, being an important framework to integrate into public health and disaster preparedness work.

The purpose of this study was to historically apply the Public Health Crisis Conceptual Model to four counties (Lincoln, Otero, Sierra, and Socorro) and their populations, including downwinders [downwinder (s) is defined as “A person who lives or has lived downwind of a nuclear test site or reactor, where the risk of being affected by radiation is greatest,” (Oxford English Dictionary n.d.)], who were most affected by the nuclear test in 1945, with a focus on community and local priorities. If the model could have been applied in 1945, what might have been different with respect to research, epidemiological studies, and compensation?

## 2. Methods

A historical descriptive case study approach (Grove and Gray 2021; Priya 2021) was used and the Public Health Crisis Conceptual Model (Figure 2) was applied to the four counties (Lincoln, Otero, Sierra, and Socorro) surrounding the Trinity test site and the population most affected by the detonation of the first nuclear weapon, including down-winders. Through the lens of community and local priorities, with a focus on responding to research, epidemiological studies, and reparations, comparisons between 1945 and the present day were conducted. A literature review and document search were conducted using the following search terms: “Alamogordo,” “Trinity test site,” “atomic bomb,” “Downwinders,” “cancer,” and combinations of these terms.

## 3. Results

**Figure 3.**
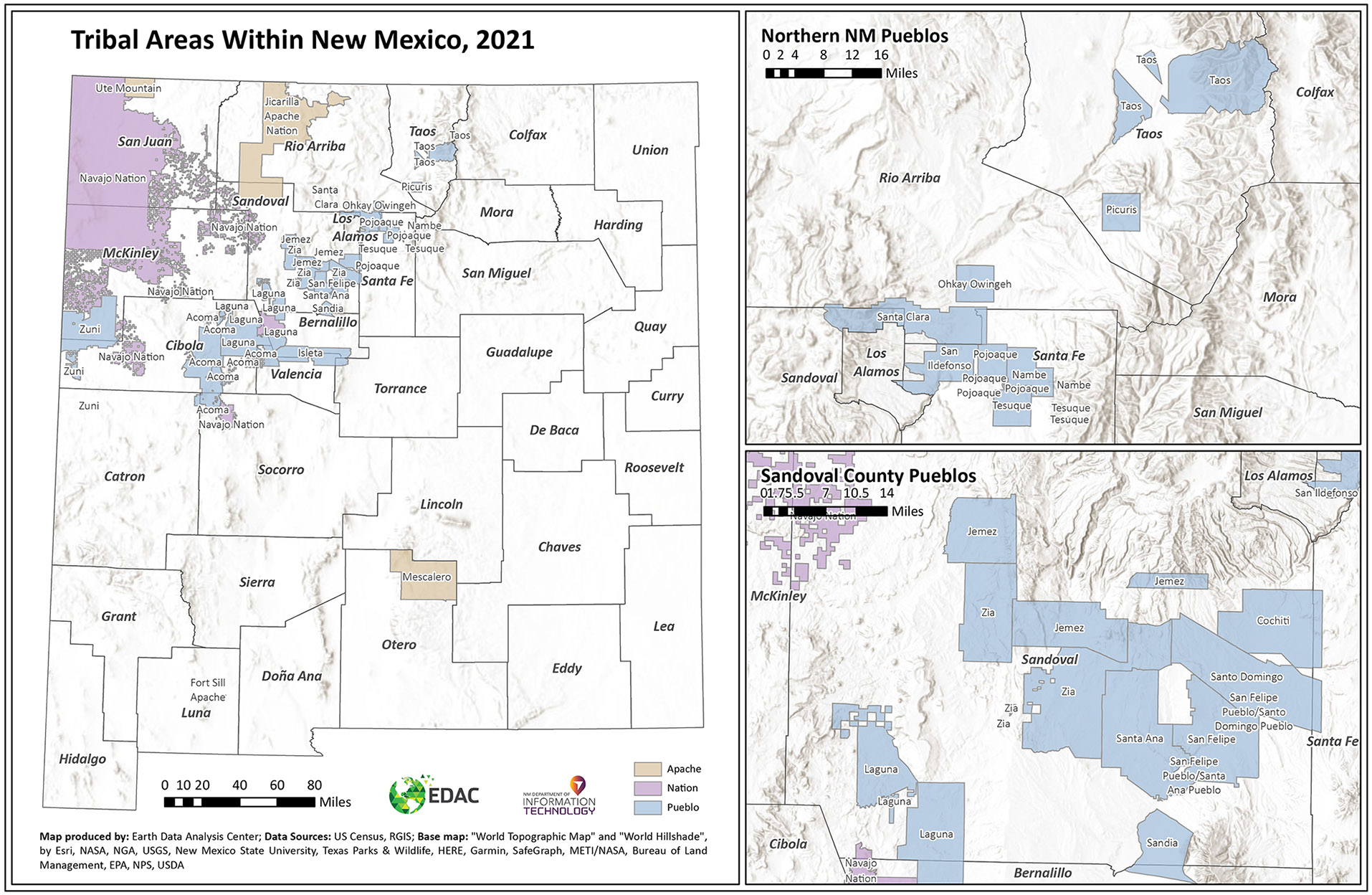
Tribal areas within New Mexico (New Mexico Department of Information Technology 2021).

New Mexico, the fifth largest state in the US, covers 121,293 square miles. Of the 33 counties in the state, seven are considered urban, and the remaining are considered rural or frontier. In 2020, the population was 2,117,522 (U.S. Census Bureau 2021). In 1940, the New Mexico census reported a population of 531,818 (U.S. Census Bureau 1940, p. 695). The state is also home to 23 Native American/Alaskan Native Tribes (New Mexico Indian Affairs Department n.d.) (Figure 3) and is composed of 50.2% Hispanic or Latino, 35.7% White (not Hispanic or Latino), 11.2% American Indian/Alaska Native (AI/AN), and fewer than 3% African American, Asian, Native Hawaiian and Other Pacific Islander, and two or more races (U.S. Census Bureau n.d.a). In 2017, the state ranked first for deaths from chronic liver disease/cirrhosis, fourth for deaths by suicide, fifth for deaths from accidents, and seventh for deaths from diabetes (Table 2).

### 3.1. Influencing Factors

**Figure 4.**
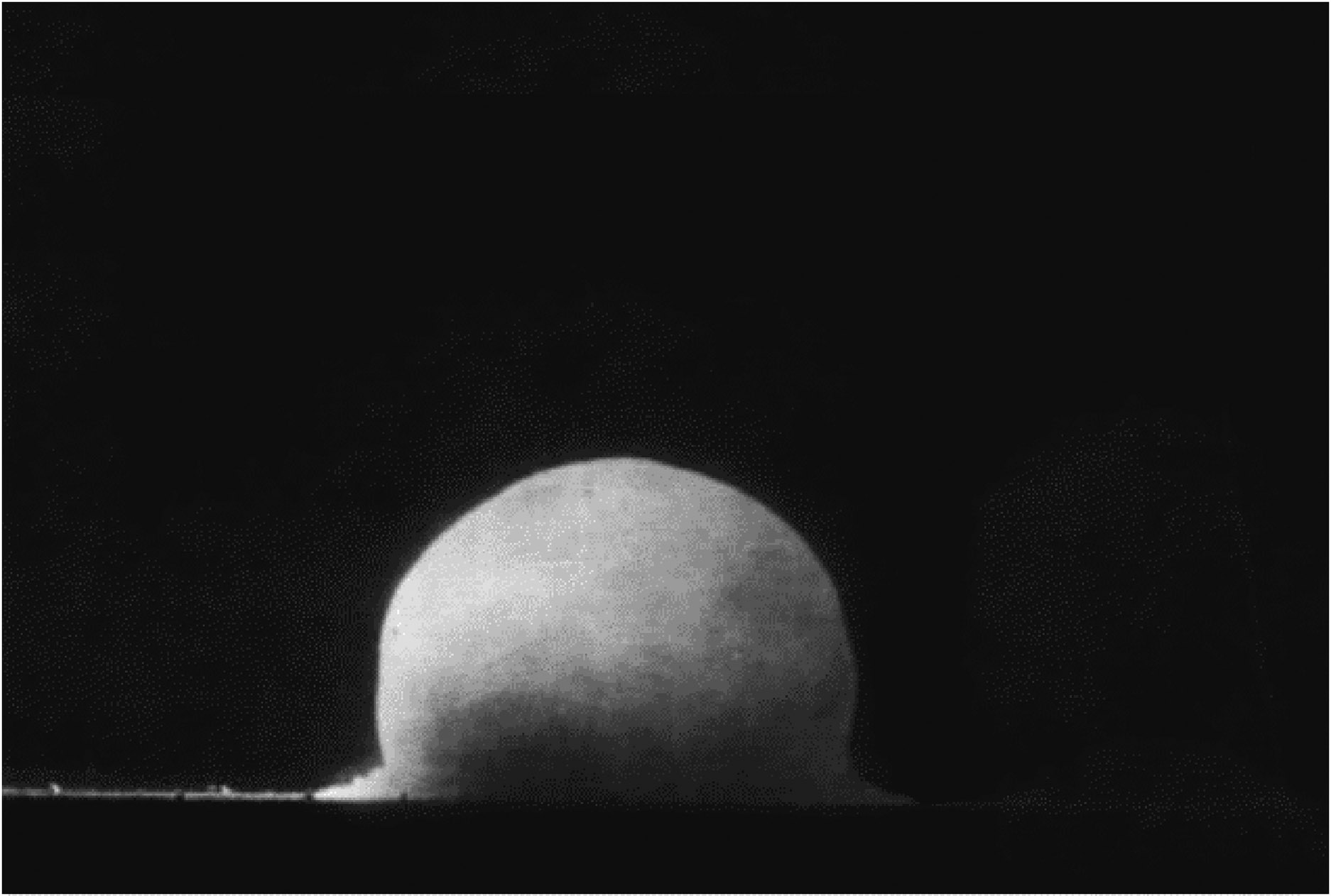
Photo of nuclear detonation at Trinity site (U.S. Department of Energy n.d.).

**Figure 5.**
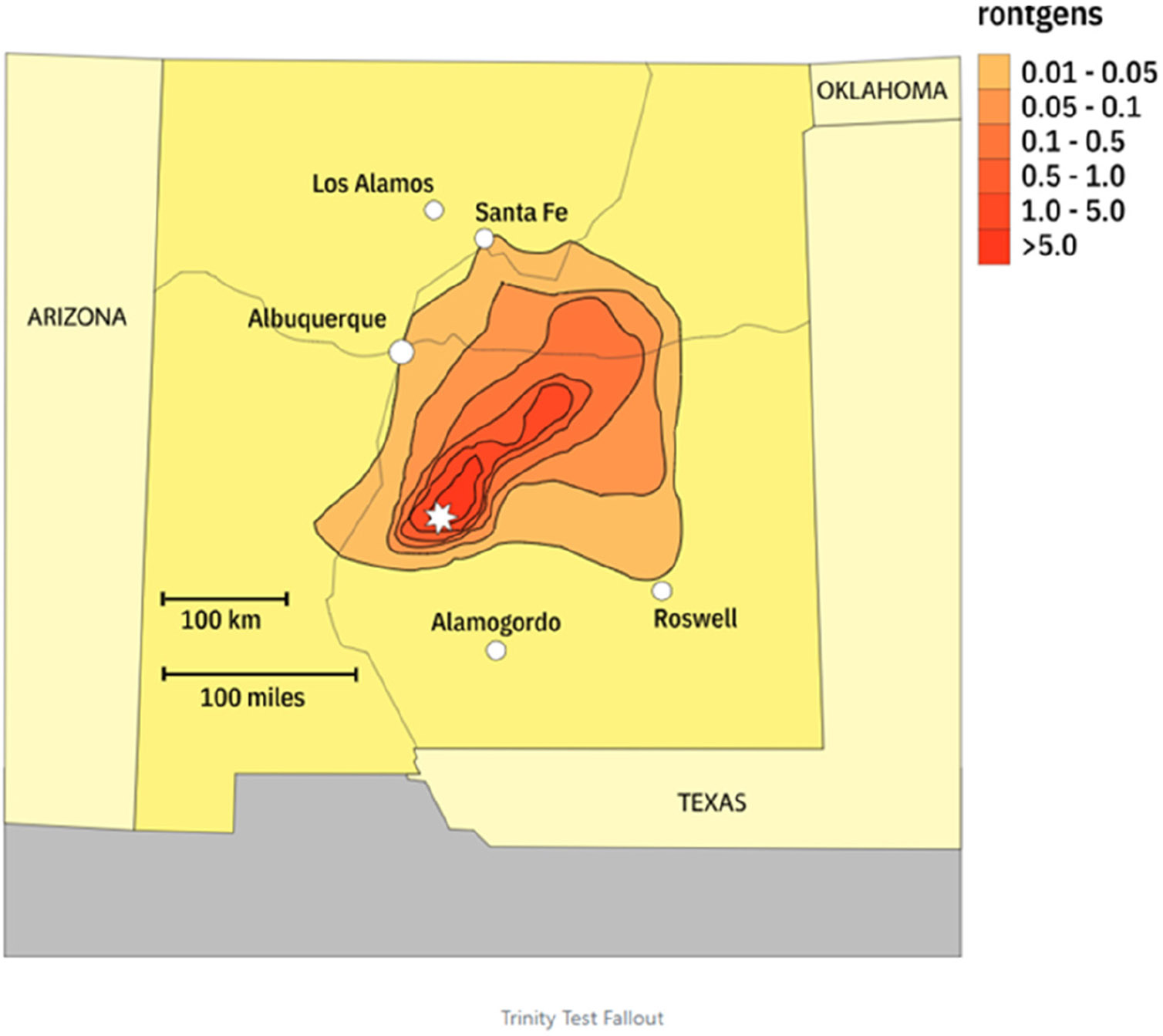
Map of Trinity test fallout (with permission from AtomicArchive.Com, sourced from Los Alamos Records). The fireball created by the explosion touched the ground and vaporized large amounts of soil. These relatively particles, highly radioactive, fell out of the cloud quickly, causing a fair amount of local fallout. Delayed or long-distance fallout was relatively small, though it was high enough to cause defects to appear on film as far away as New York. From: LA 1027-DEL

New Mexico is the site of the first nuclear explosion in 1945 at Alamogordo bombing range, which is 210 miles south of Albuquerque, NM. At the time of the blast, there were approximately 37,500 people living in the community and surrounding counties—Lincoln, Otero, Sierra, and Socorro (U.S. Census Bureau 1940). The Trinity test, a plutonium implosion device, was detonated early in the morning of 16 July 1945 (Figure 4). An estimated “18.6 kilotons of power” were discharged. The 100-foot tower that housed the bomb was evaporated, and the sand and blacktop surrounding the tower were metamor-phosized into green glass as result of the force from the detonation (US Air Force et al. n.d.). A map of the estimated fallout (ionizing radiation in roentgens), developed from Los Alamos records, shows higher radiation at the Trinity test site and lower levels in the surrounding areas (Figure 5) (Atomicarchive.com n.d.). More recently, Phillipe and colleagues (Philippe et al. 2023) published a map of the US, showing fallout from 101 nuclear tests spread out across the US.

**Figure 6.**
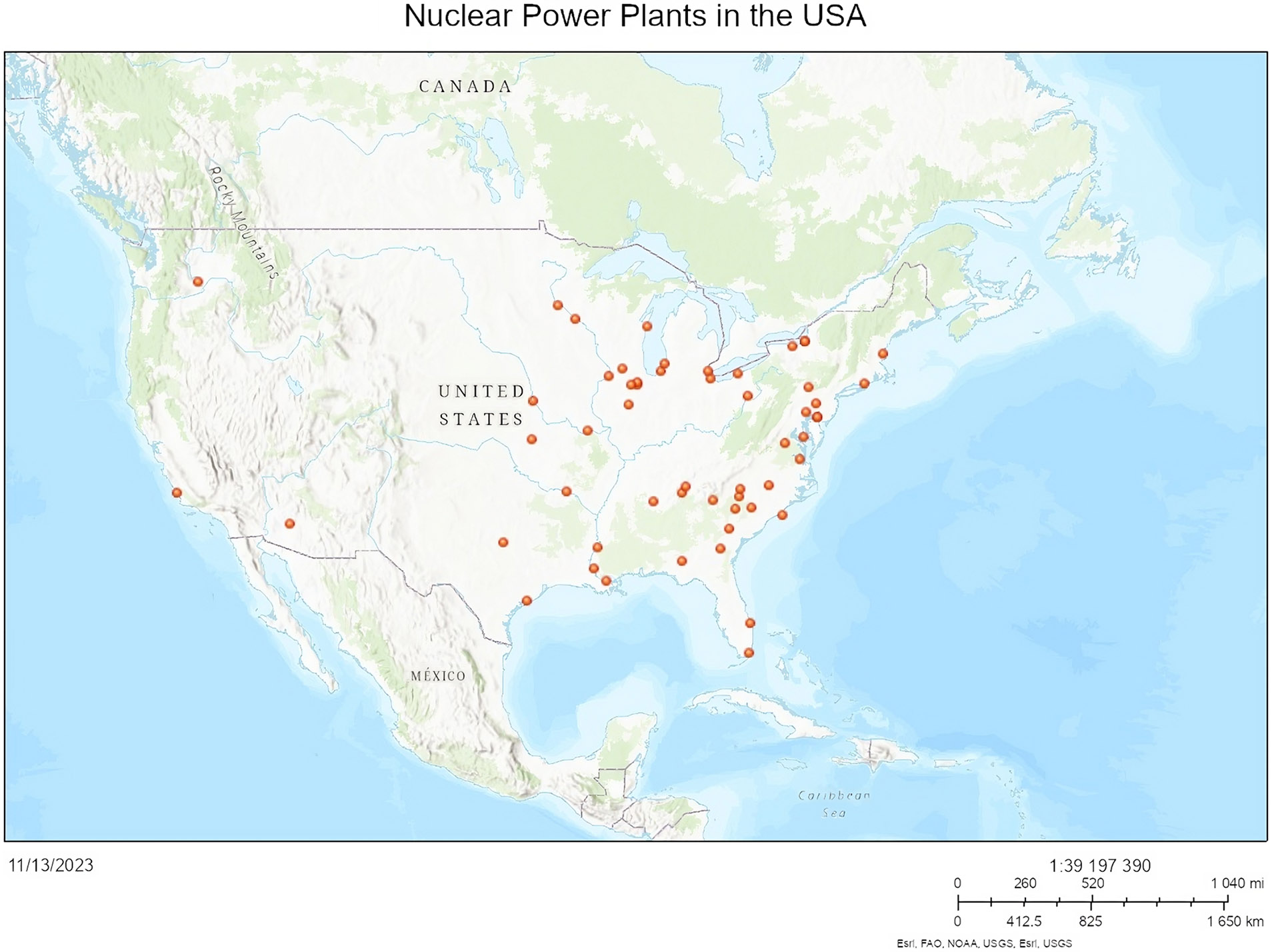
Nuclear power plants in the continental United States (Lavin and Ramos 2022).

**Figure 7.**
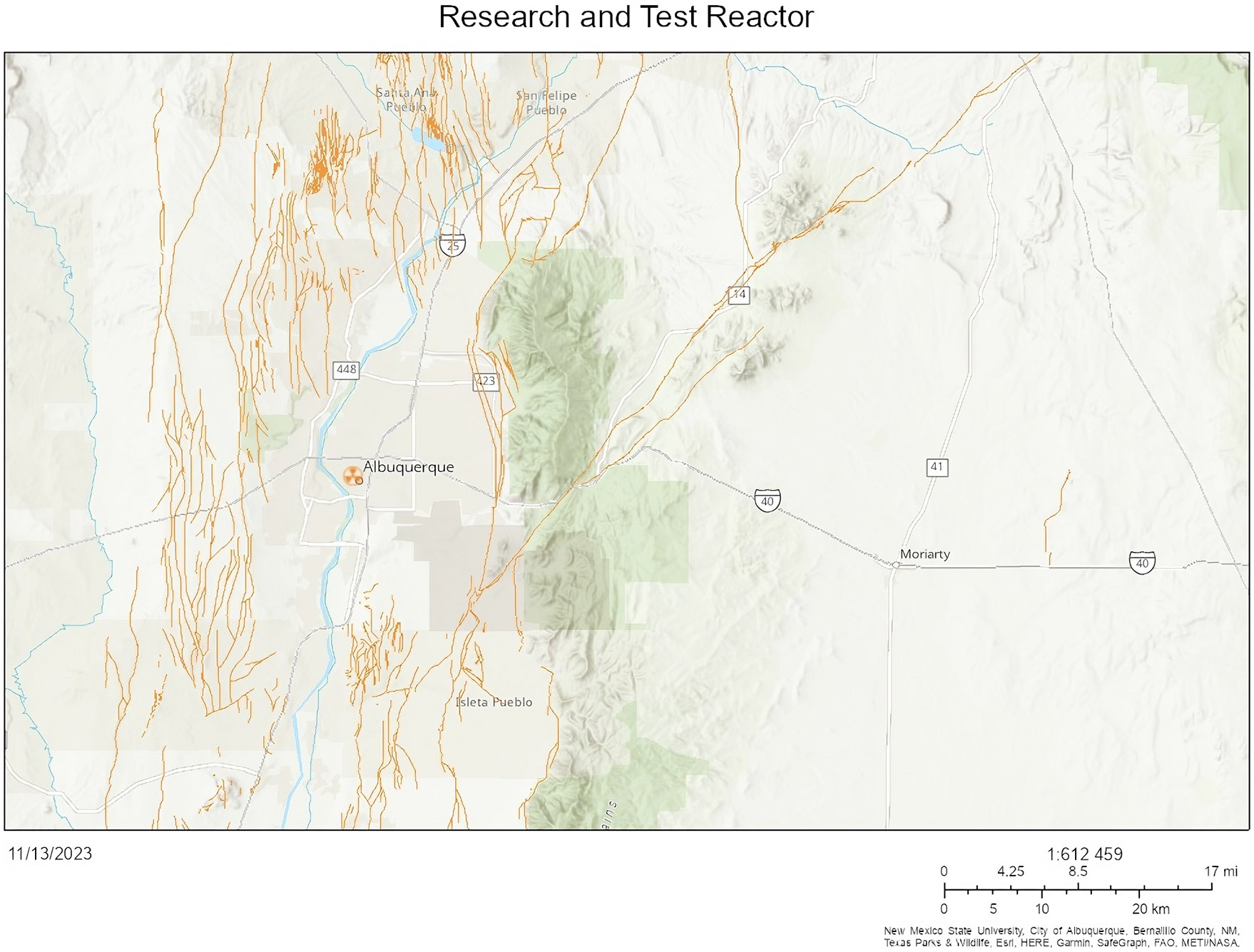
Research and test facility at the University of New Mexico (Lavin and Ramos 2022). The orange dots represent fault lines and the symbol by Albuquerque represents the operating research and test reactor at the University of New Mexico.

**Figure 8.**
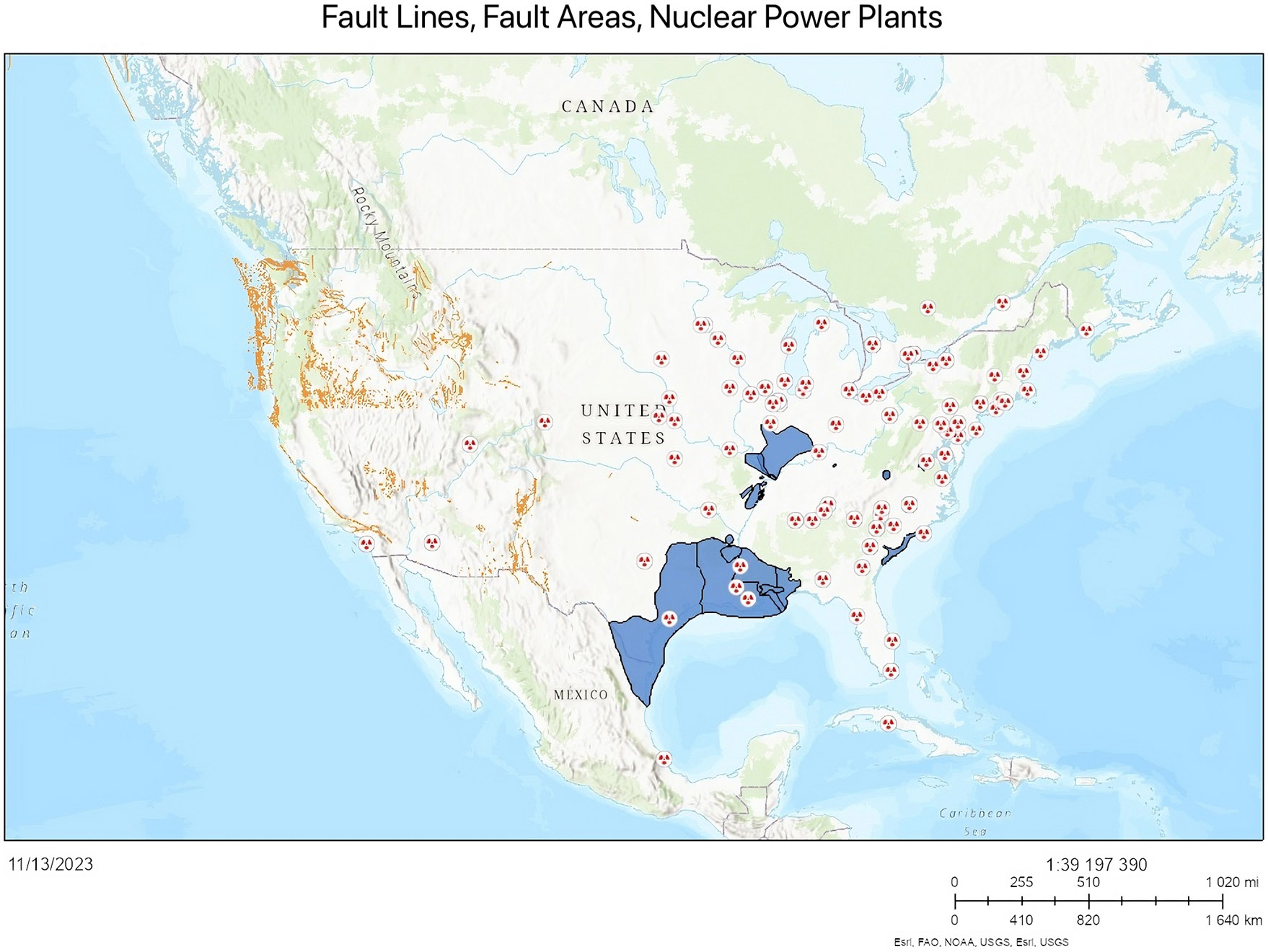
Nuclear power plants with earthquake fault lines and fault areas (Lavin and Ramos 2022). The circles with the red dots represent nuclear power plants. The orange dots represent fault lines and the blue color represents fault areas.

There are no nuclear power plants in New Mexico (Figure 6) (Lavin and Ramos 2022). There is a nuclear research and test facility at the University of New Mexico (Figure 7) (Lavin and Ramos 2022). Nuclear facilities and their proximity to earthquake fault lines are shown in (Figure 8) (Lavin and Ramos 2022). Additionally, there are fuel cycle facilities in Eunice and Hobbs, NM; a uranium recovery facility in Crown Point, NM; and three facilities undergoing decommissioning: Homestake, Rio Algom-Ambrosia Lake, and United Nuclear Corporation, near Gallup, New Mexico (U.S. Nuclear Regulatory Commission 2022).

**Figure 9.**
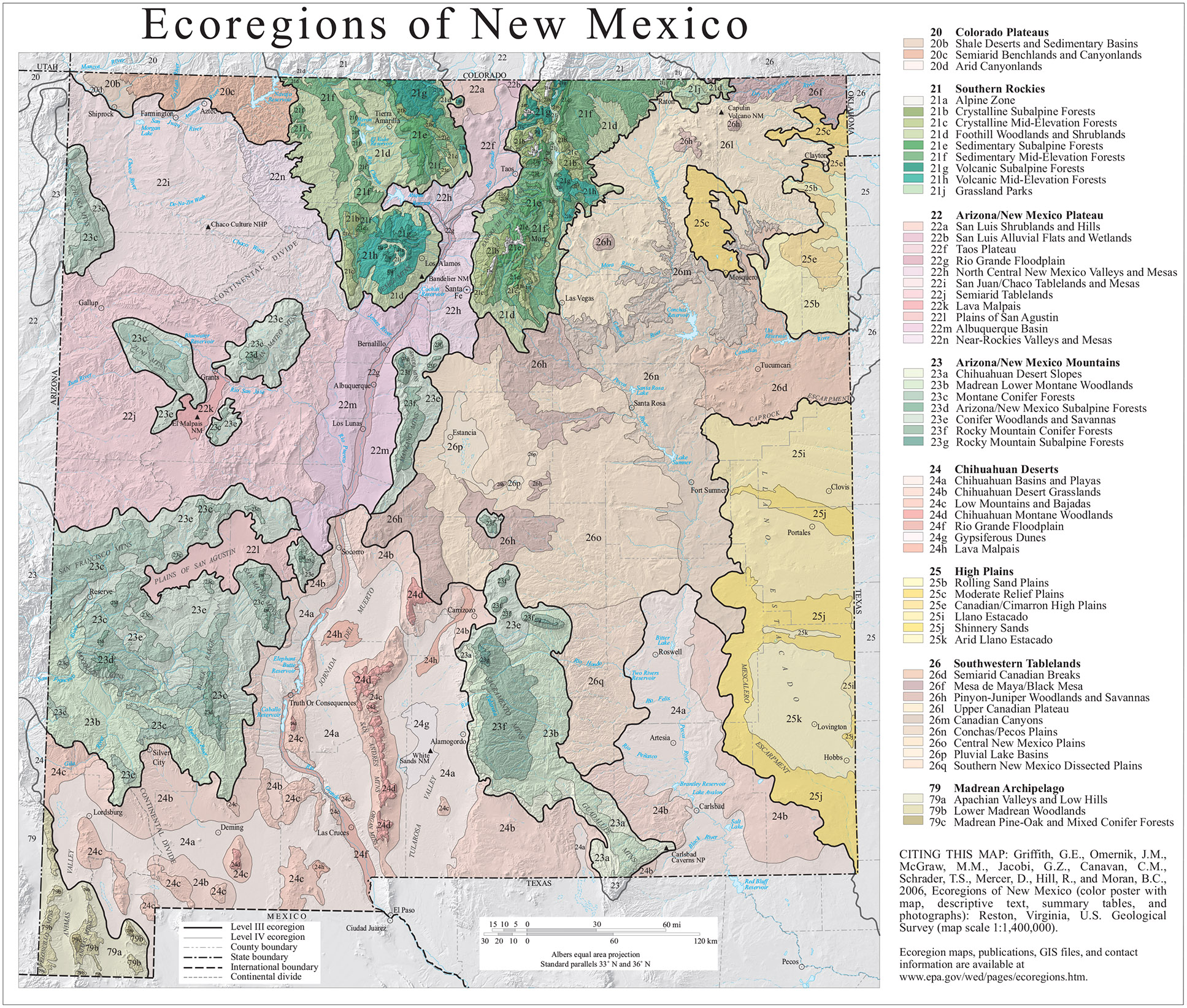
Ecoregions of New Mexico (Griffith et al. 2006).

New Mexico is divided into four land regions and eight ecoregions (Figure 9) (Griffith et al. 2006). The area for the Trinity test site was chosen for its remoteness, lack of wind, and the flat land.

### 3.2. Systems and Structures

New Mexico established the State Board of Health of New Mexico, and the first meeting was held in 1919. At that same time, the Division of Public Health Nursing was established (New Mexico Department of Health, Office of New Mexico Vital Records and Health Statistics, Public Health Division 2002). Public health nurses (PHNs) were extensively used to provide health services and education, and they were considered cost-effective given the rural nature of the state and its low population numbers. In 2002, the New Mexico Department of Health published a report (New Mexico Department of Health, Office of New Mexico Vital Records and Health Statistics, Public Health Division 2002) highlighting four areas from the *Ten Great Public Health Achievements in the United States from 1900–1999* (Centers for Disease Control and Prevention 1999). The 2002 report described the challenges of providing health services and addressing infectious diseases, clean water and sanitation, maternal and child health, and motor vehicle safety. Access to healthcare due to limited funding and the geographic features of the state created challenges in managing these health and public health issues.

The Manhattan Project and the Trinity test were kept secret, primarily to prevent other nations, such as Germany, Japan, and Russia, from learning the extent of US advances in developing an atomic bomb. In the late 1890’s, scientists advanced their knowledge of radiation and, in the first decades of the 1900’s, discovered that radiation can cause harmful effects, including changes to genes and physiologic changes that could lead to death (Environmental Protection Agency n.d.). British scientists recognized the need for protections for humans, and in 1915, the British Roentgen Society voted to “protect people from overexposure to X-rays” (Khare et al. 2014, p. 1).

The Army had systems in place to support the development of nuclear weapons and to protect the workers. The Smyth report (1945) details the administrative aspects of the project, encompassing the production of plutonium, the separation of isotopes, as well as providing a general description of working on the bomb and addressing health concerns. Chapter 7, “The Plutonium Production Problem as of February 1943,” describes “The Health Problem:”

Besides the hazards normally present during construction and operation of a large chemical plant, dangers of a new kind were expected here. Two types of radiation hazard were anticipated—neutrons generated in the pile and alpha particles, beta particles and gamma rays emitted by products of the pile. Although the general effects of the radiations had been proved to be similar to those of X-rays, very little detailed knowledge was available. Obviously the amounts of radioactive material to be handled were many times greater than had ever been encountered before.
The health group had to plan three programs: (1) provision of instruments and clinical tests to detect any evidence of exposure of the personnel; (2) research on the effects of radiation on persons, instruments, etc.; and (3) estimates of what shielding and safety measures must be incorporated in the design and plan of operation of the plant (Smyth 1945 pp. 122–23).

In November 2010, the Centers for Disease Control and Prevention (CDC) released a comprehensive report on information related to the operations, materials, material releases, residential areas, and the significance of material releases with respect to health risks from the Los Alamos National Laboratory (Los Alamos Historical Document Retrieval and Assessment Project et al. 2010). Chapter 10 is devoted to the Trinity test site. Preparations were made to monitor the fallout immediately following the detonation and evacuate the community if needed. Fallout traveled as far as Indiana, as evidenced by the contamination of the cardboard used by the Kodak Company. A company official determined that the contamination was the result of a nuclear explosion in the US but kept this information secret until 1949 (Centers for Disease Control and Prevention 2010, pp. 10–25).

### 3.3. Community and Local Priorities

Multiple sources report that those who lived in the surrounding area near the Trinity test site, including one family who lived 12 miles away, were not told about the impending test, nor were they told the truth immediately afterward (Gomez 2017; Los Alamos Historical Document Retrieval and Assessment Project et al. 2010; Smyth 1945; Tularosa Basin Downwinders Consortium n.d.b). In fact, a complete study of the radiation exposure, along with projected cancer risks, was not published until 2021 (Simon et al. 2021). Affidavits collected by the Tularosa Basin Downwinders Consortium (TBDC) are posted on their website. The affidavits illustrate the surprise about the bomb and document immediate effects from the bomb and fallout and how many family members had or have cancer (Tularosa Basin Downwinders Consortium n.d.a). As a result of not knowing about the detonation, the community was not able to provide input into the military research and monitoring, nor were they able to articulate concerns regarding potential exposure to themselves and their families from the fallout and potential contamination of water, food sources, and the environment.

The Tularosa Basin Downwinders Consortium was created in 2005 to collect data and experiences of the downwinders exposed to the fallout from the Trinity test and to advocate for compensation that others with similar exposures are eligible to receive. (Tularosa Basin Downwinders Consortium n.d.b). In the years following the detonation of the nuclear bomb, people living in the communities surrounding the test site have raised concerns about increased rates of cancer, thyroid disease, and other diseases, as well as about contaminated water, food sources, and the environment (Tularosa Basin Downwinders Consortium n.d.b).

As a result of class action lawsuits by the Navajo uranium miners and the downwinders from the Nevada nuclear weapons test site, the Congress passed the Radiation Exposure Compensation Act (RECA) on 5 October 1990 and the RECA Extension Act in 2022 (U.S. Department of Justice 2023). RECA’s purpose was to apologize and provide monetary reparations to persons who developed certain cancers and other serious diseases resulting from their exposure to radiation from “above-ground nuclear weapons tests or as a result of their occupational exposure while employed in the uranium industry during the build-up to the Cold War.” Reparations are provided to three categories of persons: uranium workers, persons present at atmospheric nuclear tests, and downwinders from the Nevada test site. New Mexican downwinders are excluded from RECA although it is difficult to ascertain why they were excluded, given the plethora of evidence that they were exposed to nuclear fallout from the Trinity test (U.S. Department of Justice 2023).

### 3.4. Data Sources

In 1945 and the years immediately following, some studies were conducted with respect to the Trinity test site and surrounding areas after the detonation of the first atomic bomb. Studies of radioactive materials and other related tests were also being conducted in other parts of the United States and in other countries. Wallace (1958) published a *Bibliography of Technical Reports on the Effects of Fallout*. In addition to general reports on the effects of radiation fallout on humans, the report includes the following:

1. 41 bibliographies;
2. 20 reports on fallout;
3. 230 reports on monitoring, composition and distribution;
4. 111 reports on effects;
5. 12 reports on protective measures and decontamination;
6. 12 reports on natural radioactivity;
7. 21 reports on tolerance to radiation.

Of the 447 reports, 17 mentioned New Mexico in the title or location. For some of the reports with New Mexico in the title, it could not be determined, from the title, if the findings were related to the Trinity test, e.g., Report 433, AFSWC-TN-56-2, issued by the Air Force Special Weapons Center, Kirtland AFB, New Mexico, *Safe Level of Contamination from Fission Products* (Dick et al. 1956) (Wallace 1958, p. 50). One report, marked secret, was from Los Alamos Scientific Laboratory. It was a *Health Physics Report on Radioactive Contamination Throughout New Mexico Following the Nuclear Explosion*, with report number 141 (Wallace 1958, p. 18).

The Los Alamos Historical Document Retrieval and Assessment (LAHDRA) project (Los Alamos Historical Document Retrieval and Assessment Project et al. 2010) was a long-term project, lasting 11 years, undertaken to conduct a comprehensive review of Los Alamos National Laboratory’s records on chemicals and radioactive materials released into the environment. Hundreds of thousands of documents were reviewed. From that group, approximately 7400 documents and 1000 drawings were summarized. With respect to the downwinders and other New Mexicans:

New Mexico residents were neither warned before the 1945 Trinity blast, informed of health hazards afterward, nor evacuated before, during or after the test. Exposure rates in public areas from the world’s first nuclear explosion were measured at levels 10,000-times higher than currently allowed. Residents reported that fallout ‘snowed down’ for days after the blast, most had dairy cows, and most collected rainwater off their roofs for drinking. All assessments of doses from the Trinity test issued to date have been incomplete in that they have not addressed internal doses received after intakes of radioactivity through inhalation or consumption of contaminated water or food products (Los Alamos Historical Document Retrieval and Assessment Project et al. 2010, pp. ES-34, 35).

Sixty-two years after the Trinity test, in 2007, the National Cancer Institute was asked by the U.S. Congress to assist with determinations on potential exposure and risks from the Trinity test. In the introduction to the manuscripts that reports on the studies that were conducted as a result of that request, Simon (2020) reports “To date, however, there has not been an assessment of public exposures and health risks from Trinity,” (Simon 2020, p. 389).

In 2023, other data sources that are publicly available provided information on multiple parameters within the Public Health Crisis Conceptual Model including the following:

1. The social determinants of health (Healthy People 2030; U.S. Department of Health and Human Services, Office of Disease Prevention and Health Promotion n.d.);
2. The seven vital conditions for health and well-being (Community Commons n.d.b);
3. County-level statistics on the composition of the population (e.g., age and sex, race and ethnicity, population characteristics, housing, family and living arrangements, education, computer access and use, transportation, and income and poverty) (U.S. Census Bureau n.d.b);
4. County health rankings and roadmaps (University of Wisconsin, Population Health Institute n.d.);
5. National health statistics (Centers for Disease Control and Prevention, National Center for Health Statistics 2023);
6. New Mexico Health and Human Services Data Book (New Mexico Human Services Department 2022).

### 3.5. Critical Decision Making

Once information was released about the bombs, critical decision-making could and should have included the development of research and epidemiological studies and the creation of a registry to monitor people who had been exposed to fallout. This was a missed opportunity to study the short and potential long-term effects of fallout with a nuclear bomb detonated close to the ground.

### 3.6. Recommendations for Education, Practice, and Planning

It is critical that health professional students, including nurses, understand the radiation risks present in New Mexico, and that they are prepared to be trusted sources of health education and community collaborators who can partner in disaster response to ensure that the impacted people have the resources they need to adapt, without major health consequences. Health professionals also need to understand how radiation contamination works so that they can be alert to potential clinical signs and symptoms of radiation exposure and the long term impacts on health. In addition, health professionals must prepare through community engagement and become a trusted source of health information and risk mitigation strategies. Communities need to plan in partnership with health professionals and the emergency management professionals responsible for identifying hazards and developing plans for mitigation, preparedness, response, and recovery. Education and training on community listening, partnership, and relationship building are critical.

## 4. Discussion

In the late 1930’s and early to mid-1940’s the United States and other countries were engaged in World War II. The United States declared war against Japan after the 7 December 1941 attack at Pearl Harbor on U.S. military bases on the island of Oahu, Hawaii. To maintain a military advantage, secrecy of the Manhattan Project was imperative (U.S. Department of Energy, Office of History and Heritage Resources n.d.). Despite the need for secrecy while the atomic bomb was under development until its first use against Japan, there was ample scientific evidence that radioactive materials could cause harm to humans.

The Smyth report (1945) documents that government officials knew of some of the dangers of radiation and expected there could be more serious consequences with larger amounts of radioactive materials (Smyth 1945 p. 41). The concern was focused on establishing a health division and developing a plan to measure exposure, as well as conducting research on the effects of radiation on both instruments and personnel (Smyth 1945 pp. 122–23). The affected communities should have been informed following the public announcements of the bombings of Hiroshima and Nagasaki and the publication of the Smyth report (Smyth 1945).

A review of the literature on radioactive fallout was the genesis for the Wallace report (1958). The review resulted in 477 reports, some designated as secret, all related to radioactive fallout—the composition, distribution and monitoring, the effects, as well as decontamination and protective procedures. The authors of these reports include the United States, Czechoslovakia, and Great Britain, the U.S. military and federal agencies, academic institutions, and private companies and laboratories. The sheer number of documents underscore the national and global interest in characterizing and understanding the effects of radioactivity and radioactive fallout in the 1950’s.

The Special Subcommittee on Radiation of the Joint Committee on Atomic Energy, Congress of the United States, Eighty-Sixth Congress, held public hearings in early May of 1959 (U.S. Congress 1959). The abstract described the extent and substantial resources devoted to a robust program of research:

… Volume 1 reviews developments since the 1957 hearings and presents new data on atmospheric and global fallout levels. It was stated that the program of fallout sampling and monitoring supported by the AEC is quite comprehensive and during 1959 approximately $2.6 million was spent on a program of scientific research with the purpose of understanding the factors that influence the patterns and rates of fallout onto the earth’s surface. This has meant sampling longitudinally, latitudinally, [sic] and vertically. A training program for scientists to carry out this program was maintained. Experimental and laboratory work centered on the movement of fallout into the food chain and water supplies and the effects of ionizing radiation on the human body and on human germ cells.

The purpose of this study was to historically apply the Public Health Crisis Conceptual Model to the four counties (Lincoln, Otero, Sierra, and Socorro) and their populations, which were most affected by the nuclear test in 1945, with a focus on community and local priorities. If the model could have been applied in 1945, what might have been different with respect to research, epidemiological studies, and compensation? The affected communities would have been informed as soon as possible after the information was made public, and epidemiologic studies, designed with input from the community to track and monitor the population, would have been implemented. New Mexicans exposed from the Trinity test would have been included in the RECA.

If the model had been applied, the Army or another designated agency would have contacted communities within the area of the greatest potential exposure to the fallout after the public announcements about the bombings of Hiroshima and Nagasaki. Using the best instrumentation, samples would have been collected and tested. The community would have been given an opportunity to participate in discussions about any proposed research and to voice their concerns about the fallout and potential contamination. Mitigation efforts for affected humans, animals, and the environment would have been planned, implemented, and monitored immediately after the detonation. Despite all of the research and projects described above, New Mexicans who were exposed to radioactive fallout from the detonation of the world’s first atomic bomb were excluded from monitoring and research studies until decades after the Trinity test. They are still excluded from reparations provided to others who were exposed to radioactive fallout despite recent efforts to include them in the Radiation Exposure Compensation Act (Cook 2023).

## 5. Limitations

For this research, information that was publicly available was used. Due to the high security related to the military mission of the Trinity test, some documents that were and still may be classified may not have been available. It is possible that relevant documents may not have been located. Whenever possible, primary or official US government documents were used as sources. The focus was deliberately narrow: on the application of the Public Health Crisis Conceptual Model, specifically examining community and local priorities in retrospect to the detonation of the first atomic bomb.

## 6. Conclusions

New Mexicans exposed to radioactive fallout from the Trinity test have not been treated equally. While there may have been justifiable reasons for the secrecy during wartime efforts, once public information had been released about the nature of the bombs detonated in Japan, everyone potentially exposed should have been notified and provided an opportunity for monitoring their health status.

Conceptual models explain systems and related concepts and can serve as a guide for research to ensure that all related components/variables are included in the development of research studies. The Public Health Crisis Conceptual Model includes community and local priorities as a foundational component of this model. Using this model should help ensure the inclusion of community input and consideration for equitable treatment of all, with a focus on those who are most vulnerable and historically marginalized. Progress has been made with respect to including communities in planning and discussions related to research; however, it is important for researchers to continue to be cognizant of including communities, especially those that are vulnerable.

## Figures and Tables

**Table 1.** Definitions for the Public Health Crisis Conceptual Model.

Concept	Definition
Access to Care	“Access to routine and critical health care is also an important factor to maintaining health and well-being.” (Community Commons n.d.a)
Amelioration or Cure	Health care providers diagnose and treat injuries and disease to ease symptoms and where possible cure conditions (Gostin 2007).
Anthropocene and Environmental Shifts (Agent, Host, Environment)	Epidemiologic principles of an external agent, a susceptible host, and a physical and social environment that brings the host and agent or vector together (Institute of Medicine 2003).
Basic Health Needs	“Physical and mental well-being starts with access to fresh air and water, nutritious food, and the security of a stable home. People also need healthy relationships-with freedom to express gender and sexuality-and a life free from violence, injury, and toxic stress.”(Community Commons n.d.a)
Community and Local Priorities	Communities must be included during all phases for their input in planning, implementing, and evaluating disaster research and other activities.
Crisis (Response)	The response phase when the goal is amelioration or cure (Lavin et al. 2012) of the human, animals, and the environment requiring critical decision making.
Effective System Coordination	Effective system coordination requires community-based disaster response capabilities and communication infrastructure. Coordination should include capacity for coordination in extenuating and changing circumstances and unknown factors should be considered, planned for and anticipated.
Flexibility and Speed	Persons adversely impacted by a disaster need immediate access to resources, even if they have lost their documentation. Previous eligibility should continue without interruption and new eligibility should be established quickly. Hence, the Federal government should endeavor to provide states and local governments with as much latitude as possible in delivering benefits equitably (Lavin and Menifee 2009).
Legal and Ethical Crisis Decision Making	The legal environment may change during a disaster and the declaration of an emergency may trigger special powers to facilitate the response through increased flexibility, limiting liability, changes in interstate healthcare licensure requirements and changes in standards of care (Hodge et al. 2009; Institute of Medicine 2009). Ethical decision-making in disaster often involves adhering to core principles, such as humanity, impartiality, neutrality, and independence (Cuthbertson and Penney 2023). These principles provide a framework to guide actions and decisions, ensuring they are ethically sound and align with professional standards and humanitarian values. Incorporating such principles into ethical decision-making helps ensure actions are morally sound and align with core values.
Postcrisis (Resilience and Recovery)	The recovery phase is when the goal is rehabilitation, maintenance of an optimal level of well-being, and support for the community and its environment. (Lavin et al. 2012). Lessons learned are key to improving education, practice, and planning.
Precrisis (Preparedness)	The preparedness phase prior to a disaster or event is a time to gather evidence and engage the community about priorities, communicate with the systems and structures, and identify influencing factors and evidence. The goal of this period is health promotion, disease prevention, and mitigation (Lavin et al. 2012).
Rapid and Rational Decision Making	Rational decision-making involves a rigorous process that relies on objective information and logical reasoning. This includes determining the issue at hand, collecting relevant data, exploring choices and potential outcomes, carefully analyzing options, examining interconnections, and, finally, selecting from among feasible options (Adam et al. 2022).
Responder Health and Safety	It is critical to protect the health and safety of disaster responders. A healthy and safe workforce is better able to help communities in response and recovery.
Self-Determination	Individuals and families impacted by a disaster have the same rights and responsibilities as everybody else. Government aid to persons adversely impacted by a disaster should therefore seek to support the self-determination of persons adversely impacted by a disaster as they seek access to public benefits and consider relocation opportunities. Individuals and families focusing on their own needs, resources, and interests are more likely to achieve favorable results for themselves and for the broader society than when government restricts or directs their choices (Lavin and Menifee 2009).
Self-Sufficiency	The object of disaster case management assistance, including efforts targeted toward persons adversely impacted by a disaster and persons with pre-disaster vulnerabilities, should be individual and family self-sufficiency. As we seek to provide every necessary benefit to help persons adversely impacted by a disaster recover from the disaster and restart their lives, the measure of our success should not be the number of new entrants into disaster assistance systems or dollars expended. Success should be measured by how quickly and successfully persons adversely impacted by a disaster and persons with pre-disaster vulnerabilities are able to become economically self-sufficient and socially integrated. These new lives may be established either in the homes and communities they occupied before the disaster or in new locations selected based on the individual’s or family’s best judgment of where their goals and aspirations may best be fulfilled (Lavin and Menifee 2009).
Systems and Structures for Health	This includes both the systems and structures for routine and emergency healthcare, mental health, and community-based and public health care. It also comprises the systems used to provide immediate care during a disaster and recovery.
Transdisciplinary Communication and Collaboration in Assessment, Planning, Implementation and Evaluation	Building systems of communication that allow for interdisciplinary collaboration and leadership models, centered on developing relationships before tasks, and including diverse insights, perspectives, values and priorities to more comprehensively understand and respond to unfolding environmental disasters should be prioritized.
Transformative Education	Health professions education, just in time training, environmental health, climate change and health and the Planetary Health Education Framework are used to inform disaster preparedness education and improve health professionals action competence in disaster preparedness, response, systems thinking and health equity.

**Table 2.** NM causes of death, 2017 (Centers for Disease Control and Prevention, National Center for Health Statistics 2018).

NM Leading Causes of Death, 2017	Deaths	Rate***	State Rank*	U.S. Rate**
1. Heart Disease	3896	151.4	32nd	165.0
2. Cancer	3620	138.3	44th	152.5
3. Accidents	1460	68.2	5th	49.4
4. Chronic Lower Respiratory Disease	1143	44.2	26th	40.9
5. Stroke	878	34.7	37th	37.6
6. Diabetes	673	26.5	7th	21.5
7. Chronic Liver Disease/Cirrhosis	605	26.8	1st	10.9
8. Alzheimer’s disease	572	22.7	32nd	31.0
9. Suicide	491	23.3	4th	14.0
10. Flu/Pneumonia	338	13.6	32nd	14.3

* Rankings are from highest to lowest.

** Rates for the U.S. include the District of Columbia and (for births) U.S. territories.

*** Death rates are age-adjusted.

## Data Availability

Data for this research can be found at the following sites: Centers for Disease Control and Prevention, National Center for Health Statistics (10 April 2018). Stats of the State of New Mexico. https://www.cdc.gov/nchs/pressroom/states/newmexico/newmexico.htm (accessed on 15 December 2023); Disaster Risk Assessment and Safety Training Interprofessional Capability (DRASTIC) Tool https://www.arcgis.com/home/item.html?id=fe701977973b451da12dc271573b20a6 (accessed on 15 December 2023). This is a compilation of multiple publicly available datasets.; U.S. Census Bureau (1940). New Mexico (p. 696). https://www2.census.gov/library/publications/decennial/1940/population-volume-1/33973538v1ch07.pdf (accessed on 15 December 2023).
